# Heart failure and tricuspid regurgitation: the role of SGLT2 inhibitors in improving outcomes: Insights from SHEBAHEART big data registry

**DOI:** 10.1093/ehjcvp/pvag018

**Published:** 2026-03-30

**Authors:** Ranel Loutati, Viana Copeland, Shir Elimeleh, David Hochstein, Kobi Faierstein, Assi Milwidsky, Sagit Ben-Zekry, Amit Segev, Rafael Kuperstein, Elad Maor

**Affiliations:** The Olga and Lev Leviev Heart Center, Chaim Sheba Medical Center Hospital - 2 Derech Sheba, Tel Hashomer, Ramat Gan 52621, Israel; School of Medicine, Tel Aviv University, Tel Aviv, Israel - Ramat Aviv, Tel Aviv 6997801, Israel; The Olga and Lev Leviev Heart Center, Chaim Sheba Medical Center Hospital - 2 Derech Sheba, Tel Hashomer, Ramat Gan 52621, Israel; School of Medicine, Tel Aviv University, Tel Aviv, Israel - Ramat Aviv, Tel Aviv 6997801, Israel; The Olga and Lev Leviev Heart Center, Chaim Sheba Medical Center Hospital - 2 Derech Sheba, Tel Hashomer, Ramat Gan 52621, Israel; School of Medicine, Tel Aviv University, Tel Aviv, Israel - Ramat Aviv, Tel Aviv 6997801, Israel; The Olga and Lev Leviev Heart Center, Chaim Sheba Medical Center Hospital - 2 Derech Sheba, Tel Hashomer, Ramat Gan 52621, Israel; School of Medicine, Tel Aviv University, Tel Aviv, Israel - Ramat Aviv, Tel Aviv 6997801, Israel; The Olga and Lev Leviev Heart Center, Chaim Sheba Medical Center Hospital - 2 Derech Sheba, Tel Hashomer, Ramat Gan 52621, Israel; School of Medicine, Tel Aviv University, Tel Aviv, Israel - Ramat Aviv, Tel Aviv 6997801, Israel; The Olga and Lev Leviev Heart Center, Chaim Sheba Medical Center Hospital - 2 Derech Sheba, Tel Hashomer, Ramat Gan 52621, Israel; School of Medicine, Tel Aviv University, Tel Aviv, Israel - Ramat Aviv, Tel Aviv 6997801, Israel; The Olga and Lev Leviev Heart Center, Chaim Sheba Medical Center Hospital - 2 Derech Sheba, Tel Hashomer, Ramat Gan 52621, Israel; School of Medicine, Tel Aviv University, Tel Aviv, Israel - Ramat Aviv, Tel Aviv 6997801, Israel; The Olga and Lev Leviev Heart Center, Chaim Sheba Medical Center Hospital - 2 Derech Sheba, Tel Hashomer, Ramat Gan 52621, Israel; School of Medicine, Tel Aviv University, Tel Aviv, Israel - Ramat Aviv, Tel Aviv 6997801, Israel; The Olga and Lev Leviev Heart Center, Chaim Sheba Medical Center Hospital - 2 Derech Sheba, Tel Hashomer, Ramat Gan 52621, Israel; School of Medicine, Tel Aviv University, Tel Aviv, Israel - Ramat Aviv, Tel Aviv 6997801, Israel; The Olga and Lev Leviev Heart Center, Chaim Sheba Medical Center Hospital - 2 Derech Sheba, Tel Hashomer, Ramat Gan 52621, Israel; School of Medicine, Tel Aviv University, Tel Aviv, Israel - Ramat Aviv, Tel Aviv 6997801, Israel

**Keywords:** Tricuspid Regurgitation, Heart Failure, SGLT2 Inhibitors

## Abstract

**Aims:**

Sodium-glucose cotransporter-2 inhibitors (SGLT2i) have transformed heart failure (HF) management, yet their effect in patients with concomitant tricuspid regurgitation (TR) remains unclear. We evaluated the association of SGLT2i use with clinical outcomes in HF patients with and without TR.

**Methods and results:**

We analysed a cohort of patients with HF (2014–2024) who underwent echocardiography within 90 days of diagnosis. Patients were stratified by significant TR and SGLT2i use. The primary outcome was a composite of all-cause mortality and HF hospitalization. TR progression was a secondary outcome. Inverse probability treatment weighting and time-dependent Cox models were used to adjust for baseline and treatment differences. Among 28 940 HF patients [median age 75 (IQR 66–83), 43% women], 4043 (14%) had significant TR, and 2320 (8%) received SGLT2i. Over a median follow-up of 3.5 years (IQR 1–7) and 79 313 echocardiograms, 11 646 (40%) patients experienced the primary outcome. Significant TR was independently associated with worse outcomes [adjusted HR (aHR) 1.21, 95% CI 1.14–1.28, *P* < 0.001] while SGLT2i use was associated with lower event rates (aHR 0.79, 95% CI 0.69–0.92; *P* = 0.002), with consistent associations across TR strata (aHR 0.65 with vs. 0.83 without significant TR; *P* for interaction = 0.180). SGLT2i was also associated with 28% reduced risk for TR progression (95% CI 0.58–0.90; *P* < 0.001). Results were consistent across HF subtypes and sensitivity analyses.

**Conclusion:**

Significant TR portends worse prognosis in HF. SGLT2i therapy is associated with improved outcomes and attenuated TR progression in this high-risk population, with findings limited by the observational design and potential residual confounding.

## Graphical Abstract

**Graphical Abstract:**
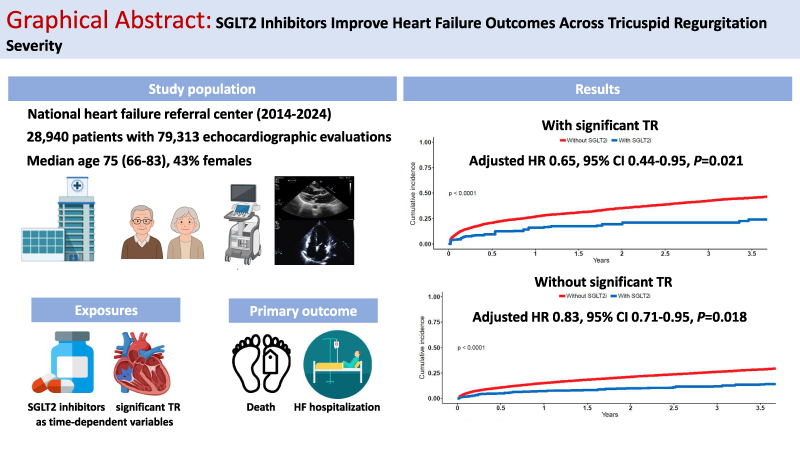
This analysis investigated the association between SGLT2i use and clinical outcomes among HF patients, with and without significant TR. Among 28 940 patients with HF, the composite outcome of death and HF hospitalization was highly prevalent, with a notably higher risk observed in those with concomitant significant TR. Treatment with SGLT2i was associated with improved clinical outcomes across both subgroups, irrespective of TR status, supporting the potential use of SGLT2i in this high-risk population. HF = Heart failure, SGLT2i = Sodium-glucose cotransporter-2 inhibitors, TR = Tricuspid regurgitation.

## Introduction

Despite advancements in treatment, heart failure (HF) remains a major burden on healthcare systems, representing the leading cause of cardiovascular hospitalizations and a key contributor to cardiovascular mortality.^1,2^ Tricuspid regurgitation (TR) is highly prevalent among HF patients, with a bidirectional relationship between the two conditions.^3,4^ In HF, TR may arise from several overlapping mechanisms, including right ventricular (RV) dilation and dysfunction, right atrial (RA) and annular dilation, or interference from cardiac implantable electronic devices (CIED), and is classified on imaging as ventricular secondary (V-STR), atrial secondary (A-STR), or CIED-associated TR.^3–5^ The presence of TR in HF patients is not benign, as evidence from registries and retrospective analyses suggests that severe TR is independently associated with poor outcomes, regardless of significant comorbidities, including HF.^6–8^

The emergence of sodium-glucose cotransporter-2 inhibitors (SGLT2i) has revolutionized the medical management of HF, demonstrating benefits across the spectrum of HF subtypes.^9,10^ These benefits range from morphological changes in the heart chambers,^11^ to anti-arrhythmic effects,^12^ and the enhancement of positive physiological mechanisms, including increased natriuresis, improved vascular resistance, haematocrit elevation, and better glycaemic control.^13,14^ However, due to the exclusion of most patients with severe valvular heart disease, including TR, from pivotal clinical trials evaluating SGLT2i in HF,^15^ data on their prognostic implications in this population are lacking.

Therefore, this study aimed to evaluate both clinical outcomes and TR progression in HF patients treated with SGLT2i, stratified by TR status, using a large, long-term HF database, with the goal of providing valuable insights into the role of this pivotal drug class in managing HF patients with concomitant significant TR.

## Methods

### Study population

This retrospective cohort study included all adult patients (>18 years) with a new clinical diagnosis of HF who visited Sheba Medical Center between January 2014 and December 2024. Patients who did not undergo a comprehensive echocardiographic evaluation, including assessment of TR severity, at our institution's laboratory within 90 days of HF diagnosis, and those who had undergone any tricuspid valve intervention before the HF diagnosis, were excluded. The analysis incorporated all echocardiograms performed during the follow-up period, totalling 79 313 echocardiograms for 28 940 unique patients (see Supplementary material online, *Figure S1*). The study was based on the SHEBAHEART big data registry that was described previously.^16^ The electronic medical records, including echocardiographic reports, served as the primary data source. The Institutional Review Board of the Sheba Medical Centre approved this study based on strict maintenance of participants’ anonymity during database analyses. No individual consent was obtained.

### Heart failure ascertainment and SGLT2 inhibitors use definition

Ascertainment of HF was based on a comprehensive review of electronic medical records documented by Sheba Medical Center providers during inpatient and outpatient visits, in accordance with European Society of Cardiology (ESC) guidelines, requiring symptoms and/or signs of HF with objective cardiac disease.^17^ HF was preidentified using the International Classification of Diseases, 9th Revision codes 404.x and 428.x, and 10th Revision code I50.x, or detected through records of HF-related treatments [e.g. chronic diuretic use, SGLT2i, angiotensin receptor neprilysin inhibitor (ARNi)], CRT implantation, reduced left ventricular ejection fraction (LVEF), diastolic dysfunction/elevated filling pressures on echocardiography, or elevated natriuretic peptides. Diagnoses were validated by cross-referencing identified records with clinical data from medical record reviews and applying logical consistency checks to detect discrepancies. Board-certified cardiologists reviewed electronic medical records to ensure the accuracy of the algorithm used to identify HF patients. Patients were dichotomized as having HF with reduced (HFrEF) or mildly-reduced/preserved ejection fraction (HFmrEF/HFpEF) based on LVEF from the first post-diagnosis echocardiogram, using a 40% cutoff. HFmrEF and HFpEF were grouped to ensure sufficient statistical power for subgroup analyses, and since both follow similar guideline-recommended management.^17^ SGLT2i treatment was defined as a documented prescription of approved agents available in Israel (dapagliflozin or empagliflozin), recorded in the electronic medical records by Sheba Medical Center providers during any clinical encounter and confirmed by patient report. Prescriptions issued outside the institution were captured only if patients reported their use during a documented visit. Medication dosing was not consistently recorded and therefore was not included in the analysis.

### Standard echocardiographic and Doppler measurements

Two-dimensional transthoracic echocardiographic and Doppler studies were obtained with state-of-the-art clinical ultrasound machines equipped with 3.5-MHz transducers using standard views and performed by specialized sonographists. The Sheba Medical Center echocardiography laboratory is a tertiary laboratory, staffed with academic board-certified cardiologists, all of whom completed echocardiography fellowships in North America. TR was categorized as none, trivial, mild, mild-to-moderate, moderate, moderate-to-severe, and severe using an integrative, semi-quantitative approach.^16,18^ This approach encompassed the following: Assessment of colour Doppler jet area, tricuspid valve morphology, RA and RV size, inferior vena cava (IVC) diameter and respiratory variation, hepatic venous flow reversal, jet density, and contour of continuous wave Doppler envelope, aligning with the guidelines’ recommendations.^19^ For this analysis, TR was considered significant if it was at least moderate in severity. Patients with significant TR were hierarchically classified into one of four aetiological categories. Primary TR was assigned to patients with echocardiographic evidence of intrinsic tricuspid valve apparatus damage. The V-STR group included patients with systolic pulmonary artery pressure (sPAP) ≥ 40 mmHg and/or left heart disease (LHD), defined as LVEF ≤ 40% or the presence of significant left-sided valvular disease, as well as those with reduced RV function or enlargement. The CIED-associated TR group comprised patients with an RV lead but without evidence of pulmonary hypertension, LHD, or RV dysfunction/enlargement. Finally, cases were classified as A-STR if they did not meet the criteria for the other groups and at least one parameter associated with A-STR phenotype.^16,20^ All reported echocardiographic measurements, including LV diameters, volumes, mass, ejection fraction, diastolic function, LA diameter, RV size, area, and systolic function, as well as RA pressure and area, were evaluated in accordance with established standards.^21,22^ The presence of significant valvular heart disease other than TR, including aortic stenosis (AS), mitral regurgitation (MR), and mitral stenosis (MS), was also assessed and reported following the latest guidelines’ criteria.^21^

### Clinical data and study endpoint

Baseline demographic and clinical data, including laboratory results, comorbidities, and medication use, were obtained from electronic patient records. Diagnoses relied on hospitalization records (ICD-9/10 codes), laboratory tests, medications, physiological signals (e.g. ECGs), radiological images (e.g. echocardiograms), and procedure reports. Data accuracy, including diagnoses, laboratory results, and medication use, was verified through selected patient case reviews. Overall, missing values were present in less than 3% of the cohort. For these cases, we used simple imputation by replacing missing values of numeric variables with the median and missing values of categorical variables with the mode. The primary outcome of this study was a composite of HF hospitalization, as adjudicated by the treating physician, and all-cause mortality. Notably, HF hospitalizations were captured only within our centre, while mortality data were obtained from national records. Progression of TR by ≥2 severity grades and worsening of sPAP by ≥5 mmHg were evaluated as secondary outcomes among patients who had at least one additional echocardiogram during follow-up, performed ≥1 month after the baseline study. The 5 mmHg threshold was considered clinically meaningful based on the standard 5 mmHg increments used for estimated right atrial pressure and the average age-related change in sPAP.^23^ Survival data was available for all subjects from the Israeli Population Register up to 31 December 2024.

### Statistical analysis

Continuous variables were expressed as mean ± standard deviation if normally distributed or median with interquartile range if skewed. Categorical variables were presented as frequency (%). Continuous data were compared using the Student's *t*-test or Wilcoxon test, as appropriate, and categorical data were compared with the use of the chi-square test or Fisher's exact test, where appropriate. For time-to-event analysis, patients were censored only in the event of death or tricuspid valve intervention, and the time-to-event was calculated from the date of the initial echocardiographic evaluation. The probability of the composite outcome according to the study groups was graphically displayed using the method of Kaplan–Meier, with a comparison of cumulative incidence across strata by the log-rank test. Univariable and multivariable Cox proportional hazards regression modelling were used to compare patients who were under SGLT2i use to patients who were not, with adjustments made for parameters that were found to be significant in the univariable model or are recognized to be associated with poorer prognosis among individuals with HF. The adjusted Cox model included age, sex, obesity (BMI ≥ 30 kg/m^2^), arterial hypertension, diabetes mellitus, ischaemic heart disease, atrial fibrillation, chronic obstructive pulmonary disease, chronic kidney disease (eGFR < 45 mL/min/1.73 m^2^), LVEF ≤ 40%, ≥ moderate AS, ≥ moderate MR, grade ≥ 2 diastolic dysfunction, elevated sPAP (≥ 40 mmHg), RV dysfunction, use of loop diuretics, thiazides, mineralocorticoid receptor antagonists (MRA), beta-blockers (BB), angiotensin-converting enzyme inhibitors (ACEi)/angiotensin II receptor blockers (ARB), angiotensin receptor neprilysin inhibitors (ARNi), glucagon-like peptide-1 (GLP-1) agonists, laboratory values within one month of diagnosis including serum sodium, potassium, haemoglobin, and the year of echocardiographic evaluation. To capture the changes in HF treatment over time and the additional data from subsequent echocardiographic evaluations, our main analyses used a time-dependent approach enabling all covariates, including SGLT2i use and TR status, to be accountable even if not existed at baseline. Patients contributed person-time to the non-SGLT2i group from the index echocardiogram until SGLT2i initiation and to the SGLT2i group thereafter, minimizing immortal time bias by ensuring that pre-treatment survival time was not attributed to the treated group. Patients who initiated SGLT2i before the index date contributed all follow-up time to the SGLT2i group, whereas those who never initiated SGLT2i contributed all time to the non-SGLT2i group. Because SGLT2i were not randomly assigned, inverse probability treatment weighting (IPTW) was applied to mitigate confounding by indication. Propensity scores were estimated using logistic regression, incorporating baseline characteristics that were significantly different between groups and clinically relevant variables listed in *Table 1*. To account for temporal trends in patient characteristics and treatment strategies, a mid-period stratification variable was added to the model. The inverse of the propensity score and its complement (1 − propensity score) were used as weights for treated and untreated patients, respectively, with extreme weights excluded. To prevent artificial inflation of the sample size, stabilized IPTW was applied, ensuring weighted estimates of descriptive characteristics. Interaction analysis was performed with significant TR as a categorical variable to assess whether concomitant TR modifies the association between SGLT2i use and improved outcomes in HF patients. Subgroup analysis by HF type, in which HFmrEF and HFpEF were grouped together to ensure adequate statistical power, was performed. A subgroup of patients with predominant right-sided HF phenotype, defined as HFmrEF/HFpEF without elevated sPAP, was also evaluated. In addition, multiple sensitivity analyses were conducted to assess the robustness and generalizability of our findings, each excluding patients with specific clinical characteristics or echocardiographic features. A one-year landmark analysis was also performed. Lastly, a 1:1 propensity score matching (PSM) analysis was conducted using the same covariates apllied in the IPTW approach, and associations with the composite outcome were assessed through a time-dependent Cox model, consistent with the primary analyses. All analyses were performed using R software version 4.3.3 (R Foundation for Statistical Computing). An association was considered statistically significant for a two-sided *P* value of less than 0.05.

**Table 1:** Baseline characteristics by SGLT2i use

	All (*n* = 28 940)	No SGLT2i (*n* = 26 620)	SGLT2i (*n* = 2320)	*P* value
Age, years	75 (66–83)	75 (66–83)	72 (64–78)	<0.001
Female sex	12 420 (42.9%)	11 716 (44%)	704 (30.3%)	<0.001
BMI, kg/m^2^	27.4 (24.3–31.3)	27.3 (24.3–31.2)	28 (24.8–32)	<0.001
Obesity (BMI ≥ 30 kg/m^2^)	8448 (29.2%)	7660 (28.8%)	788 (34%)	<0.001
HTN	15 122 (52.3%)	13 725 (51.6%)	1397 (60.2%)	<0.001
DM	9154 (31.6%)	7814 (29.4%)	1340 (57.8%)	<0.001
IHD	11 702 (40.4%)	10 465 (39.3%)	1237 (53.3%)	<0.001
HFrEF	10 316 (35.7%)	9232 (34.7%)	1084 (46.7%)	<0.001
AF	7426 (25.7%)	6830 (25.7%)	596 (25.7%)	0.993
COPD	2535 (8.8%)	2354 (8.8%)	181 (7.8%)	0.096
CKD (eGFR < 45 mL/min/1.73m_2_)	6893 (23.8%)	6504 (24.4%)	389 (16.8%)	<0.001
CVA	4132 (14.3%)	3761 (14.1%)	371 (16.0%)	0.015
Malignancy	2750 (9.5%)	2535 (9.5%)	215 (9.3%)	0.714
Anti-coagulations	7875 (27.2%)	7136 (26.8%)	739 (31.9%)	<0.001
Insulin	3617 (12.5%)	3009 (11.3%)	608 (26.2%)	<0.001
Oral anti-diabetics	8536 (29.5%)	6937 (26.1%)	1599 (68.9%)	<0.001
Beta blockers	19 070 (65.9%)	17 282 (64.9%)	1788 (77.1%)	<0.001
Loop diuretics	20 815 (71.9%)	19 130 (71.9%)	1685 (72.6%)	0.445
Thiazide diuretics	2628 (9.2%)	2410 (9.1%)	218 (9.4%)	0.607
MRA	5957 (20.6%)	5194 (19.5%)	763 (32.9%)	<0.001
ACEi/ARB	16 090 (55.6%)	14 579 (54.8%)	1511 (65.1%)	<0.001
ARNi	423 (1.5%)	231 (0.9%)	192 (8.3%)	<0.001
GLP-1 agonists	525 (1.8%)	311 (1.2%)	214 (9.2%)	<0.001
CCB	9297 (32.1%)	8576 (32.2%)	721 (31.1%)	0.27
Statins	16 321 (56.4%)	14 614 (54.9%)	1707 (73.6%)	<0.001
Serum Na	139 (136–141)	139 (136–141)	139 (137–141)	<0.001
Serum K	4.2 (3.9–4.6)	4.2 (3.9–4.6)	4.3 (4–4.7)	<0.001
Haemoglobin	11.8 (10.3–13.2)	11.7 (10.3–13.2)	12.4 (10.8–13.9)	<0.001

Values are no.(%) or median (IQR). ACEi = Angiotensin Converting Enzyme inhibitors; AF = Atrial Fibrillation; ARB = Angiotensin Receptor Blockers; ARNi = Angiotensin Receptor Neprilysin inhibitors; BMI = Body Mass Index; CCB = Calcium Channel Blockers; COPD = Chronic Obstructive Pulmonary Disease; CVA = Cerebro-Vascular Accident; DM = Diabetes Mellitus; eGFR = estimated Glomerular Filtration Rate; GLP-1 = Glucagon-Like Peptide-1; HFrEF = Heart Failure with reduced Ejection Fraction; HTN = Hypertension; IHD = Ischaemic Heart Disease; IPTW = Inverse Probability Treatment Weighted; MRA = Mineralocorticoid Receptor Antagonist; SGLT2i = Sodium-Glucose coransporter-2 inhibitors

## Results

Final study population comprised 28 940 patients, with a median age of 75 years [interquartile range (IQR): 66–83], of whom 12 420 (43%) were female. Sixty-four per cent of HF patients had HFmrEF/HFpEF (*n* = 18 624). At the time of HF diagnosis, 2320 patients (8%) were treated with SGLT2i, including 1322 (57%) on empagliflozin and 998 (43%) on dapagliflozin. Compared to those not receiving these medications, SGLT2i-treated patients were younger (median age 72 vs. 75 years, *P* < 0.001), more often male (70% vs. 56% male, *P* < 0.001), and had a higher prevalence of obesity (34% vs. 29%, *P* < 0.001), arterial hypertension (52% vs. 60%, *P* < 0.001), diabetes mellitus (29% vs. 58%, *P* < 0.001), ischaemic heart disease (39% vs. 53%, *P* < 0.001), and HFrEF (35% vs. 47%, *P* < 0.001). Conversely, they had a lower rate of chronic kidney disease (17% vs. 24%, *P* < 0.001). Use of glucose-lowering agents, statins, and guideline-directed HF therapies, including BB, MRA, ACEi/ARB, and ARNi, was also significantly more common among those receiving SGLT2i (*Table 1*). After IPTW, baseline characteristics were well balanced between groups, with minimal residual differences that, when present, often shifted direction compared to the unweighted analysis (see Supplementary material online, *Table S1*).

All patients in the study population had their TR status evaluated at baseline. In the original cohort, 4043 (14%) patients had significant TR at baseline; among these, 283 (7%) were treated with SGLT2i at the time of their first echocardiographic evaluation. In comparison to those without significant TR, patients with significant TR were older (median age 80 vs. 74 years, *P* < 0.001), mostly female (57% vs. 41%, *P* < 0.001), and less frequently obese (24% vs. 30%, *P* < 0.001). While the prevalence of reduced LVEF and diastolic dysfunction was similar between the groups, those with significant TR had higher rates of concomitant valvular heart disease, larger left and right atrial dimensions, elevated sPAP, and higher rates of RV dysfunction, as detailed in Supplementary material online, *Table S2*. At baseline, the majority of significant TR cases were classified as V-STR [*n* = 3072 (76%)], followed by A-STR [*n* = 641 (16%)], primary TR [*n* = 242 (6%)] and CIED-associated TR [*n* = 88 (2%)]. The use of SGLT2i was most common among patients with V-STR [*n* = 230 (7.5%)], and similar between those with A-STR [*n* = 36 (5.6%)], primary TR [*n* = 12 (5%)] and CIED-associated TR [*n* = 5 (5.7%)].

### Mortality and heart failure hospitalizations during follow-up

**Figure 1:**
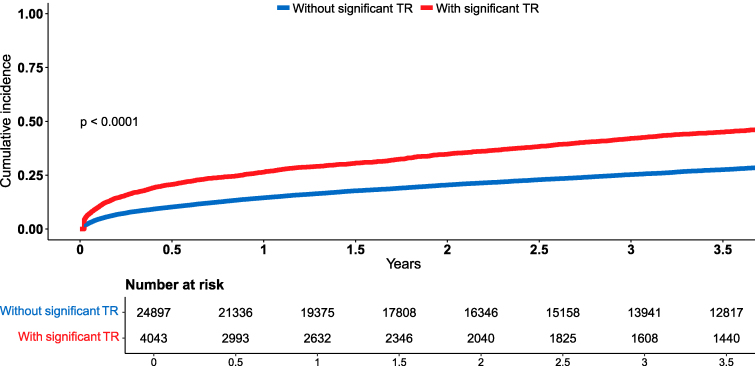
Kaplan-meier curves for the cumulative incidence of the primary outcome stratified by baseline TR status. Kaplan-Meier cumulative incidence curves of the composite outcome in the study population before applying IPTW, demonstrating a higher incidence of mortality or HF hospitalization among patients with HF and significant TR. Log rank *P* < 0.001. HF = Heart Failure; TR = Tricuspid Regurgitation.

During a median follow-up period of 3.5 years (IQR: 1–7), with a median of 2 (IQR: 1–3) echocardiographic assessments per patient, and a total of 79 313 performed echocardiograms, 11 646 (40%) patients experienced the primary composite outcome (3752 [13%] HF hospitalizations and 8763 [30%] deaths). This included 2123 (53%) outcomes among those with significant TR vs. 9523 (38%) outcomes among those without. Kaplan-Meier analysis revealed that the cumulative probability of the composite outcome at the median follow-up of 3.5 years was 45.4%±0.8% among patients with significant TR vs. 27.8%±0.3% in patients without significant TR (*Figure 1*; *P* Log rank < 0.001 for the overall difference during follow-up). In unadjusted Cox regression, significant TR at baseline was associated with an 84% increased risk of the composite outcome (95% CI 1.75–1.92, *P* < 0.001), with an independent association observed in the multivariable analysis (HR 1.21, 95% CI 1.14–1.28, *P* < 0.001). Additional independent predictors of the composite outcome are presented in Supplementary material online, *Table S3*.

### SGLT2 inhibitors use, tricuspid regurgitation status, and composite outcome

**Figure 2:**
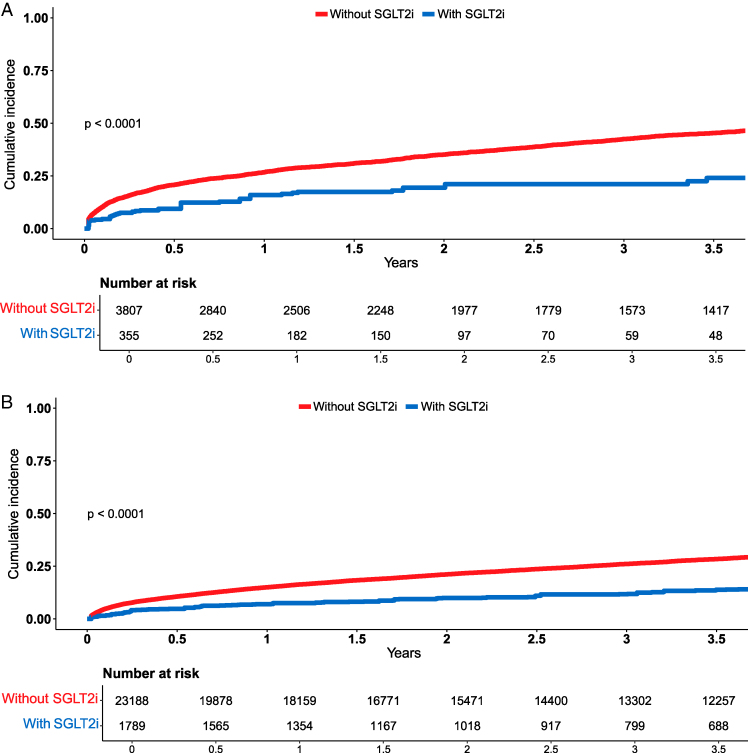
Kaplan-meier curves for the cumulative incidence of the primary outcome by baseline SGLT2i use in patients with and without significant TR. Kaplan-Meier cumulative incidence curves of the composite outcome in the pseudopopulation after applying IPTW, demonstrating that the association between SGLT2i use and lower risk for mortality or HF hospitalization does not depend on TR status. A: In patients with significant TR (*n* = 4156), the incidence of the composite utcome was 24% vs. 45.7% in SGLT2i treated vs. untreated patients. B: In patients without significant TR (*n* = 24 872), the corresponding rates were 13.8% vs. 28.6%. Log rank *P* < 0.001 for both. HF = Heart Failure; SGLT2i = Sodium-Glucose Cotransporter-2 inhibitors; TR = Tricuspid Regurgitation.

At baseline, crude event rates of the composite outcome were 16.8% (391/2320) among patients treated with SGLT2i, compared to 42.3% (11 255/26 620) among those not receiving this therapy. The association between baseline SGLT2i use and the risk of death or HF hospitalization in the unweighted cohort is given in Supplementary material online, *Table S3*. After applying IPTW, SGLT2i use at baseline remained significantly associated with a reduced risk of the primary composite outcome in both unadjusted and adjusted Cox models (*Table 2*). This association was consistent across subgroups stratified by TR status, as demonstrated by Kaplan-Meier analyses (*Figures 2A* and *B*; *P* < 0.001 for both log-rank tests), and confirmed in subgroup-specific multivariable Cox models (*Table 2*; *P* for interaction = 0.36).

**Table 2:** Multivariable Cox regression estimates for the primary outcome associated with SGLT2i use, stratified by TR status

	All (HR, 95% CI, *P* value)	Without significant TR (HR, 95% CI, *P* value)	With significant TR (HR, 95% CI, *P* value)
Baseline analysis			
HF hospitalization	HR 0.48, 95% CI 0.31–0.74, *P* < 0.001	HR 0.49, 95% CI 0.30–0.82, *P* = 0.007	HR 0.42, 95% CI 0.19–0.93, *P* = 0.032
All-cause mortality	HR 0.63, 95% CI 0.51–0.78, *P* < 0.001	HR 0.68, 95% CI 0.54–0.86, *P* < 0.001	HR 0.46, 95% CI 0.29–0.74, *P* = 0.001
Composite outcome	HR 0.59, 95% CI 0.48–0.72, *P* < 0.001	HR 0.62, 95% CI 0.50–0.77, *P* < 0.001	HR 0.50, 95% CI 0.33–0.75, *P* < 0.001
Time-dependent analysis			
HF hospitalization	HR 0.64, 95% CI 0.53–0.77, *P* < 0.001	HR 0.67, 95% CI 0.56–0.80, *P* = 0.001	HR 0.53, 95% CI 0.35–0.91, *P* = 0.028
All-cause mortality	HR 0.84, 95% CI 0.72–0.97, *P* = 0.022	HR 0.88, 95% CI 0.75–1.02, *P* = 0.091	HR 0.71, 95% CI 0.49–1.03, *P* = 0.071
Composite outcome	HR 0.79, 95% CI 0.69–0.92, *P* = 0.002	HR 0.83, 95% CI 0.71–0.95, *P* = 0.018	HR 0.65, 95% CI 0.44–0.95, *P* = 0.021

Cox estimates are from multivariable model on the baseline and time-dependent data after IPTW, adjusted for the following variables: age, sex, obesity, arterial hypertension, diabetes mellitus, ischaemic heart disease, atrial fibrillation, chronic obstructive pulmonary disease, chronic kidney disease, LVEF < 40%, ≥ moderate AS and ≥ moderate MR, ≥ grade 2 diastolic dysfunction, elevated sPAP, RV dysfunction, loop diuretics, thiazides, MRA, BB, ACEi/ARB, ARNi, GLP-1 agonists, serum Na, serum K, haemoglobin, and the year of echocardiographic evaluation.

HF = Heart Failure; SGLT2i = Sodium-Glucose Cotransporter-2 inhibitors; TR = Tricuspid Regurgitation

During follow-up, a total of 79 313 echocardiographic evaluations were performed. SGLT2i therapy, initiated either at baseline or during follow-up, was documented in 4200 patients (14.5%), including 2562 (61%) treated with empagliflozin and 1638 (39%) with dapagliflozin (see Supplementary material online, *Figure S2*). Of them, 496 (11.8%) had significant TR at baseline. In the main time-dependent analysis, the use of SGLT2 was associated with a 24% lower risk of death or HF hospitalization, compared to HF patients not receiving SGLT2i (unadjusted HR 0.76, 95% CI 0.66–0.87, *P* < 0.001). This association remained significant after multivariable adjustment (adjusted HR 0.79, 95% CI 0.69–0.92, *P* = 0.002). Overall, significant TR was observed in 5994 unique patients (20.7%), of whom 928 (15.5%) received SGLT2i. Univariable Cox analysis, incorporating SGLT2i use and significant TR as time-dependent covariates, showed consistent results such that patients treated with SGLT2i had a lower risk of the composite outcome compared to untreated patients, irrespective of TR status (HR 0.63, 95% CI 0.42–0.93, *P* = 0.014 for patients with TR; HR 0.77, 95% CI 0.67–0.90, *P* < 0.001 for patients without TR; *P* for interaction = 0.4). In a multivariable Cox model with time-dependent covariates, the association between SGLT2i use and reduction in the risk of the composite outcome remained consistent, without significant interaction between TR status and SGLT2i use, indicating that the effect of SGLT2i extends to HF patients with significant TR (HR 0.65, 95% CI 0.44–0.95, *P* = 0.021 for patients with TR; HR 0.83, 95% CI 0.71–0.95, *P* = 0.018 for patients without TR; *P* for interaction = 0.18). Analyses of the individual components of the composite outcome indicated that the observed associations were primarily explained by a reduction in HF hospitalizations (*Table 2*).

### TR progression, sPAP worsening, and SGLT2 inhibitors use

A follow-up echocardiogram with assessment of sPAP and TR status, performed ≥1 month after the baseline study, was available in 14 679 patients (50.7%). Among them, 899 (6.1%) were receiving SGLT2i at baseline, and 2779 (18.9%) received SGLT2i at any point during follow-up. Significant TR was present in 1879 patients (12.8%) at baseline. Of these, 95 (5%) were on SGLT2i before the first follow-up study and 276 (14.7%) before the last. TR progression, defined as an increase of ≥2 grades on the TR severity scale, and sPAP worsening, defined as a rise of ≥5 mmHg, were evaluated as secondary outcomes. During a median follow-up time of 3 years (IQR: 0.8–6.5), TR progression occurred in 2398 patients (16.3%), including 231 (9.6%) treated with SGLT2i. The distribution of TR severity at first and last echocardiographic assessments by SGLT2i use is shown in Supplementary material online, *Figure S3*. In univariable time-dependent Cox regression, SGLT2i use was associated with a nearly two-fold lower risk of TR progression (HR 0.53, 95% CI 0.42–0.68, *P* < 0.001), which remained significant after multivariable adjustment (HR 0.72, 95% CI 0.58–0.90, *P* < 0.001). Worsening of pulmonary pressures was observed in 5183 patients (35.3%), with 355 (6.8%) receiving SGLT2i. Supplementary material online, *Figure S4* presents the change in sPAP between initial and final evaluations stratified by SGLT2i use. SGLT2i therapy was associated with a 45% lower risk of sPAP worsening in univariable analysis (HR 0.55, 95% CI 0.45–0.67, *P* < 0.001), and a 36% lower risk after multivariable adjustment (HR 0.64, 95% CI 0.53–0.77, *P* < 0.001). Both TR progression and sPAP worsening were independently associated with a higher risk of death or HF hospitalization (HR 1.83, 95% CI 1.64–2.05, *P* < 0.001 for TR progression; HR 1.50, 95% CI 1.40–1.61, *P* < 0.001 for sPAP worsening).

### Subgroup analysis by HF type, multiple sensitivity analyses, and PSM analysis

Subgroup analysis by HF type as well as multiple sensitivity analyses were conducted to evaluate whether the association between SGLT2i use and clinical outcomes was modified by specific patient characteristics. The study cohort was stratified into HFmrEF/HFpEF [LVEF > 40%; *n* = 18 624 (64%)] and HFrEF [LVEF ≤ 40%; *n* = 10 316 (36%)] groups, with significant TR observed in 13.8% (2562/18 624) and 14.4% (1481/10 316) of patients, respectively. In multivariable Cox models, similar to the main analysis, SGLT2i use was associated with reduced rates of HF hospitalizations and mortality in both HF subtypes, irrespective of TR status. Among patients with HFmrEF/HFpEF, the adjusted HRs were 0.67 (95% CI 0.40–0.95, *P* = 0.035) for those with TR, and 0.81 (95% CI 0.66–0.99, *P* = 0.042) for those without TR (*P* for interaction = 0.4). Similarly, in patients with HFrEF, the corresponding HRs were 0.56 (95% CI 0.32–0.99, *P* = 0.044), and 0.82 (95% CI 0.70–0.94, *P* = 0.025) (*P* for interaction = 0.38). Restricting the analysis to patients with a predominant right-sided HF phenotype [*n* = 13 031 (45%)] produced similar results. In those without significant TR, SGLT2i therapy was associated with a comparable reduction in risk (HR 0.76, 95% CI 0.59–0.99, *P* = 0.044), with a directionally similar association in patients with significant TR (HR 0.69, 95% CI 0.36–1.05, *P* = 0.055).

**Figure 3:**
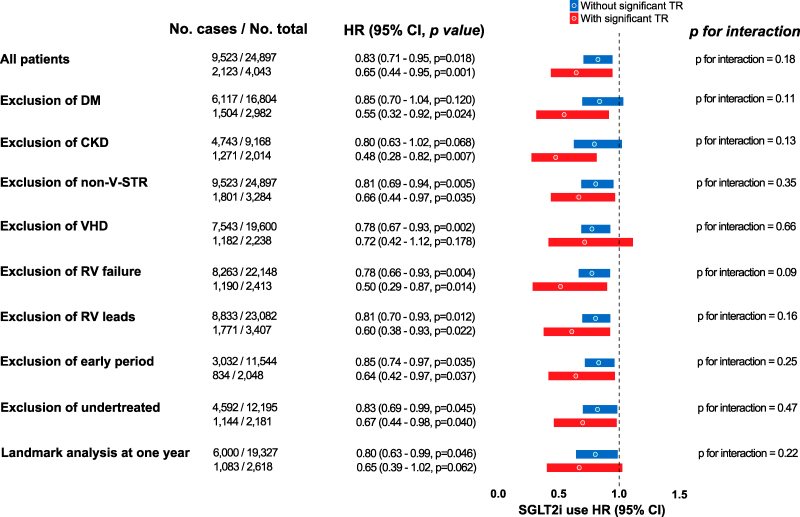
Forest plot of hazard ratios for SGLT2i use in patients with and without significant TR across multiple sensitivity analyses. This forest plot presents the hazard ratios and corresponding 95% confidence intervals for the association between SGLT2i use and the primary composite outcome, stratified by the presence (blue) or absence (red) of significant TR in multiple sensitivity analyses and one-year landmark analysis. Absolute event rates are shown, and all estimates originate from a multivariable time-dependent model. The findings demonstrate that overall, SGLT2i use is associated with a consistent reduction in risk of HF hospitalization and mortality among patients with and without significant TR despite the exclusion of key subgroups, with various degrees of interaction, in a multivariable model. Undertreated patients are defined as HF patients who received fewer than two medications, including BB, MRA, and ACEi/ARB/ARNi. CKD = Chronic Kidney Disease; DM = Diabetes Mellitus; HF = Heart Failure; HTN = Hypertension; RV = Right Ventricle; SGLT2i = Sodium-Glucose Cotransporter-2 inhibitors; TR = Tricuspid Regurgitation; VHD = Valvular Heart Disease; V-STR = Ventricular-Secondary TR.

To assess the robustness of our findings, we conducted multiple sensitivity analyses, each performed after separately excluding patients with diabetes mellitus, chronic kidney disease, non-V-STR aetiologies, significant valvular heart disease other than TR, RV dysfunction, RV pacing leads, those evaluated prior to the midpoint of the study period, and patients who were undertreated (i.e. received fewer than two HF medications, including BB, MRA, and ACEi/ARB/ARNi). A one-year landmark analysis was also performed. Results of all analyses are summarized in *Figure 3*.

Lastly, a 1:1 PSM analysis was conducted, resulting in a matched cohort of 4640 patients with balanced baseline characteristics (see Supplementary material online, *Table S4*), half of whom received SGLT2i at baseline. In this matched cohort, a time-dependent Cox model showed that SGLT2i use was associated with a 30% reduction in the composite outcome (HR 0.70, 95% CI 0.53–0.94, *P* = 0.018). This association was consistent across patients with and without significant TR (HR 0.64, 95% CI 0.35–0.98, *P* = 0.041; HR 0.71, 95% CI 0.51–0.97, *P* = 0.034, respectively), with no significant interaction (*P* for interaction = 0.65).

## Discussion

This study provides novel insights into the potential therapeutic role of SGLT2i in patients with heart failure and concomitant significant TR, representing the first investigation to examine their prognostic impact in this high-risk population and addressing a key gap in current evidence. Leveraging a large, contemporary cohort of 28 940 comprehensively characterized HF patients, including 79 313 echocardiographic assessments and detailed clinical, laboratory, and sonographic data, we conducted a longitudinal analysis at a national referral centre in Israel over a median follow-up of 3.5 years. Consistent with prior reports, we confirmed that significant TR is associated with worse outcomes in HF. Importantly, we evaluated the effect of both baseline and time-dependent SGLT2i use across TR severity, demonstrating a consistent association with lower event rates in patients with and without significant TR, and across HF subtypes. SGLT2i use was associated with reduced risk for TR progression and attenuation of pulmonary pressure elevation among patients with follow-up echocardiography, which may partially relate to the observed clinical benefit. To enhance the robustness and generalizability of our findings, we conducted multiple sensitivity analyses to address potential confounders and replicated the main analysis in a 1:1 propensity score-matched cohort.

TR and HF share a complex and reciprocal relationship. TR may result from elevated pulmonary pressures, RV failure, RA myopathy, and enlargement of the tricuspid annulus, which are often consequences of LV dysfunction and increased LV filling pressures, both characteristic features of HF.^4,24^ In turn, TR can contribute to worsening HF by reducing RV stroke volume and promoting progressive RV enlargement due to chronic volume and pressure overload. It may also indirectly impair LV function through ventricular interdependence and abnormal RV-LV interaction.^25,26^ Additionally, data from large registries have shown that TR is independently associated with worse clinical outcomes in patients with HF.^6–8^ In a contemporary cohort of 13 469 HF patients, Heitzinger et al.^7^ reported that over one-third had at least moderate secondary TR, which was significantly associated with increased mortality across all HF subtypes, independent of major clinical confounders. Despite the high prevalence and adverse prognostic implications of TR, only 3% of affected patients underwent surgical or transcatheter intervention during the study period, underscoring the need for effective medical therapies in this clinically vulnerable population. In our expanded cohort of HF patients with a median of two echocardiographic evaluations per individual, we observed that significant TR was independently associated with a 21% higher rate of HF hospitalization and mortality compared to those without TR, irrespective of comorbid conditions and consistent across both baseline and follow-up assessments. In addition, we found that SGLT2i use was associated with a lower risk of adverse outcomes among patients with coexisting significant TR, with no significant interaction between TR status and treatment effect. SGLT2i use was also associated with less TR progression and attenuation of pulmonary pressure increase over time, suggesting that these agents may modify the natural history of TR in HF patients.

The development of transcatheter therapies for TR has brought renewed attention to the management of patients with significant TR. Although procedural advances have shown safety and improvements in quality-of-life,^27–29^ consistent reductions in mortality have not been demonstrated, underscoring the need for improved patient selection.^30^ Additionally, a substantial proportion of patients with TR are not candidates for invasive intervention due to anatomical or clinical constraints,^31^ reinforcing the importance of optimizing medical therapy in this population.^3,4^ Moreover, individuals with severe TR or other significant valvular heart disease were excluded from major HF trials evaluating SGLT2i, and existing evidence does not clarify the role of these agents in HF patients with concomitant advanced TR. Our study addresses this gap by demonstrating an independent association between SGLT2i use and improved clinical outcomes in HF patients, regardless of TR status, supporting the need for further evaluation of SGLT2i as a potentially accessible, non-invasive component of guideline-directed therapy in this population.

Although SGLT2i are not yet routinely indicated for valvular heart disease, emerging evidence suggests potential benefits in patients with MR and AS. The Ertugliflozin for Functional Mitral Regurgitation (EFFORT) trial demonstrated that one year of ertugliflozin therapy significantly reduced regurgitant volume, LA volume index, and improved LV global longitudinal strain in patients with functional MR.^32^ Complementing these findings, the Dapagliflozin Effect on Functional Mitral Regurgitation and Myocardial Remodeling (DEFORM) trial showed that three months of dapagliflozin therapy led to reductions in MR severity and improvements in LVEF and other LA parameters.^33^ Collectively, these results suggest a potential therapeutic role for SGLT2i in functional MR. In individuals with non-severe AS, SGLT2i have been proposed as a potential disease-modifying therapy after a target trial emulation study demonstrated an association between their use and a slower progression to severe AS.^34^ Additionally, in patients with HF and AS undergoing transcatheter aortic valve implantation (TAVI), a randomized trial by Raposeiras-Roubin et al.^35^ showed that dapagliflozin initiation within 14 days of hospital discharge significantly reduced HF hospitalizations and all-cause mortality. Given that most patients enrolled in transcatheter tricuspid trials were not receiving SGLT2i,^27–29^ and in light of the growing evidence supporting SGLT2i benefits across valvular conditions, our findings demonstrating reduced TR progression and improved outcomes in HF patients with significant TR highlight the unique and potentially underrecognized role of SGLT2i in this population. These results contribute to an evolving view of SGLT2i as a therapeutic class with relevance beyond traditional HF management, extending into the integrated treatment of valvular heart disease.

SGLT2i exert their cardioprotective effects through a range of mechanisms, including structural, electrical, and hormonal pathways, as well as cellular-level effects such as anti-inflammatory and anti-fibrotic actions.^11–14^ In the context of valvular heart disease, and TR in particular, it is plausible that a key mechanism by which SGLT2i improve clinical outcomes involves natriuresis and osmotic diuresis. This effect is a direct consequence of SGLT2 inhibition in the proximal renal tubule, leading to glycosuria and increased urinary output.^13,14^ An additional potential mechanism underlying the observed benefits of SGLT2i in HF patients with TR is suggested by our finding that SGLT2i use was associated with a 28% lower risk of TR progression and a 36% lower risk of sPAP worsening. Similar improvements have been reported in two studies involving patients with HFrEF, in which initiation of SGLT2i therapy was associated with reductions in both sPAP and TR grade.^36,37^ Moreover, the Empagliflozin Evaluation by Measuring Impact on Hemodynamics in Patients With Heart Failure (EMBRACE-HF) trial demonstrated that empagliflozin significantly reduced pulmonary arterial pressures in patients with HF, irrespective of EF,^38^ regardless of EF, further supporting the suggested mechanism. Correspondingly, a recent meta-analysis reported that SGLT2i therapy was associated with lower sPAP and improved RV systolic function, including higher tricuspid annular plane systolic excursion values.^39^ When considered alongside our subgroup findings in patients with predominant right-sided HF, these results further support the potential role of SGLT2i in mitigating RV dysfunction and slowing TR progression.

Although our dataset did not allow for assessment of arrhythmia burden or rhythm control approaches, it is reasonable to hypothesize, based on prior literature, that SGLT2i may reduce atrial remodelling and pulmonary pressures in a manner that could complement catheter ablation strategies in patients with HF, AF, and TR.^40^

### Study strengths and limitations

This study has several notable strengths. It leverages a large, real-world cohort of unselected HF patients with comprehensive clinical and echocardiographic data, evaluated longitudinally over a follow-up period of up to 10 years. The early implementation of electronic health records at our institution enabled robust data collection across multiple time points. In Israel, up until 2024, transcatheter tricuspid interventions were not reimbursed and surgery was rarely performed, contributing to the high prevalence of medically managed TR which enables extended observation of the natural history of this condition. The use of IPTW and multivariable adjustment for key comorbidities, echocardiographic parameters, and concurrent HF therapies helped reduce confounding by indication. Frequent imaging and clinical assessments also enabled both baseline and time-dependent analyses, as well as subgroup evaluations by HF subtype, multiple sensitivity analyses, and PSM analysis, supporting the validity and generalizability of the findings.

However, several limitations should be acknowledged. The retrospective single-centre design introduces inherent biases, including potential referral bias, and HF hospitalizations were captured only within our centre, whereas mortality was ascertained nationally, which may have led to underreporting of hospitalizations and potential bias in the composite endpoint. The absence of key clinical and laboratory parameters, such as signs of right HF, NYHA functional class, natriuretic peptide levels, and information on SGLT2i dosing, adherence, and clinical contraindications, limits the comprehensiveness of the analysis.

Although TR severity was assessed using an integrative multiparametric approach, the inclusion of patients over a 10-year period resulted in inconsistent availability of quantitative indices of TR severity and RV function, including EROA, TAPSE, and S’, and data on loading conditions and volume status were not consistently recorded. These limitations, together with unassessed interobserver variability, restrict the interpretation of our secondary analyses of TR progression and sPAP worsening and should be considered when drawing mechanistic conclusions. In addition, the study was underpowered for subgroup analyses in patients with primary TR, A-STR, and CIED-associated TR, and therefore the findings are predominantly relevant to V-STR in HF patients which constituted 76% of cases. Moderate and severe TR were combined because only a small number of severe TR cases received SGLT2i, which remains an important area for future investigation given the differing clinical course and prognosis of severe TR.

The extended inclusion period spanned a decade in which HF management evolved significantly, particularly regarding the expansion of SGLT2i indications from diabetes to heart failure and chronic kidney disease. This temporal heterogeneity may have introduced bias and complicates efforts to isolate the independent effect of SGLT2i therapy. While IPTW, multivariable adjustment, and time-dependent modelling helped mitigate confounding, residual confounding remains possible as patients receiving SGLT2i may represent a healthier or more closely monitored subgroup. A sensitivity analysis excluding undertreated patients further supported the robustness of the findings, demonstrating a 17% risk reduction among patients without significant TR at baseline and a 33% reduction among those with significant TR (*P* for interaction = 0.47).

Although a time-dependent Cox model was employed to reduce biases related to treatment initiation during follow-up, the potential for immortal time bias remains, as patients must survive long enough to receive SGLT2i therapy. This was partially addressed by a complementary one-year landmark analysis, which showed consistent results for patients without significant TR and similar point estimates, albeit with wider confidence intervals, for patients with significant TR.

Finally, despite the application of advanced statistical techniques, causal inference is inherently limited by the observational study design, and randomized controlled trials are ultimately required to establish causality and guide clinical management.

### Conclusions and clinical implications

In this study, we provide new insights into the potential benefits of SGLT2i among patients with and without significant TR, using a large longitudinal cohort with up to 10 years of follow-up. Our findings suggest that SGLT2i use is associated with a lower risk of adverse outcomes across TR severity and with attenuated TR progression. However, given the observational design and the possibility of residual confounding, these results should be interpreted as associative rather than causal. These observations support further investigation of SGLT2i in this patient population and warrant confirmation in prospective randomized studies.

## Supplementary Material

pvag018_Supplementary_Data

## Data Availability

All requests for raw and analysed data and related materials will be reviewed by Sheba Medical Center’s legal department to verify whether the request is subject to any confidentiality obligations.
