# Supplementary material for: Heart failure and tricuspid regurgitation: the role of SGLT2 inhibitors in improving outcomes: Insights from SHEBAHEART big data registry
Source: Eur Heart J Cardiovasc Pharmacother. 2026 Mar 30;12(3):186–97. doi: 10.1093/ehjcvp/pvag018 (PMC13185744; doi:10.1093/ehjcvp/pvag018)
Supplement: pvag018_Supplementary_Data [file pvag018_Supplementary_Data.docx]

Supplementary Material for Manuscript:

**Heart Failure and Tricuspid Regurgitation: The Role of SGLT2 Inhibitors in Improving Outcomes**

Insights from SHEBAHEART big data registry

Supplementary Table 1 – Baseline characteristics by SGLT2i use after IPTW:

|  | All (N=29,139) | No SGLT2i (N=26,995) | SGLT2i (N=2,144) | p value | SMD |
| --- | --- | --- | --- | --- | --- |
| Age, years | 75 (66 - 83) | 75 (66 - 83) | 75 (67 - 83) | 0.398 | 0.015 |
| **Female sex** | 12,733 (43.7%) | 11,775 (43.6%) | 958 (44.7%) | 0.351 | 0.021 |
| BMI, kg/m^2^ | 27.4 (24.3 - 31.3) | 27.4 (24.3 - 31.3) | 27.4 (24.3 - 31.6) | 0.311 | 0.036 |
| **Obesity (BMI ≥ 30 kg/m**^2^**)** | 8,514 (29.2%) | 7,848 (29.1%) | 666 (31.1%) | 0.185 | 0.032 |
| **HTN** | 15,089 (51.8%) | 13,973 (51.8%) | 1,116 (52.1%) | 0.733 | 0.006 |
| **DM** | 8751 (30%) | 8,102 (30%) | 649 (30.3%) | 0.813 | 0.006 |
| **IHD** | 11,517 (39.5%) | 10,722 (39.7%) | 795 (37.1%) | 0.821 | 0.054 |
| **HFrEF** | 10,251 (35.2%) | 9,489 (35.2%) | 762 (35.5%) | 0.017 | 0.008 |
| **AF** | 7,454 (25.6%) | 6,916 (25.6%) | 538 (25.1%) | 0.609 | 0.012 |
| **COPD** | 2,545 (8.7%) | 2,376 (8.8%) | 169 (7.9%) | 0.158 | 0.033 |
| **CKD (eGFR < 45 mL/min/1.73m^2^)** | 7,123 (24.4%) | 6,541 (24.2%) | 582 (27.1%) | 0.036 | 0.051 |
| **CVA** | 4,148 (14.2%) | 3,824 (14.2%) | 324 (15.1%) | 0.24 | 0.027 |
| **Malignancy** | 2,766 (9.5%) | 2,566 (9.5%) | 200 (9.3%) | 0.817 | 0.006 |
| **Anti-coagulations** | 7,801 (26.8%) | 7,273 (26.9%) | 528 (24.6%) | 0.021 | 0.053 |
| **Insulin** | 3,373 (11.6%) | 3,193 (11.8%) | 180 (8.4%) | <0.001 | 0.114 |
| **Oral anti-diabetics** | 7,751 (26.6%) | 7,285 (27.0%) | 466 (21.7%) | <0.001 | 0.123 |
| **Beta blockers** | 19,041 (65.3%) | 17,626 (65.3%) | 1,415 (66.0%) | 0.525 | 0.015 |
| **Loop diuretics** | 21,047 (72.2%) | 19,415 (71.9%) | 1,632 (76.1%) | <0.001 | 0.096 |
| **Thiazide diuretics** | 2,639 (9.1%) | 2,439 (9.0%) | 200 (9.3%) | 0.677 | 0.010 |
| **MRA** | 5,812 (19.9%) | 5,415 (20.1%) | 397 (18.5%) | 0.09 | 0.039 |
| **ACEi/ARB** | 16,037 (55.0%) | 14,839 (55.0%) | 1,198 (55.9%) | 0.429 | 0.018 |
| **ARNi** | 359 (1.2%) | 355 (1.3%) | 4 (0.2%) | <0.001 | 0.122 |
| **GLP-1 agonists** | 484 (1.7%) | 477 (1.8%) | 7 (0.3%) | <0.001 | 0.142 |
| **CCB** | 9,430 (32.4%) | 8,677 (32.1%) | 753 (35.1%) | 0.005 | 0.063 |
| **Statins** | 16,025 (55.0%) | 14,934 (55.3%) | 1,091 (50.9%) | <0.001 | 0.089 |
| **Serum Na** | 139 (136 - 141) | 139 (136 - 141) | 138 (136 - 141) | 0.005 | 0.053 |
| **Serum K** | 4.2 (3.9 - 4.6) | 4.2 (3.9 - 4.6) | 4.2 (3.9 - 4.6) | 0.322 | 0.012 |
| **Hemoglobin** | 11.7 (10.3 - 13.2) | 11.7 (10.3 - 13.2) | 11.6 (9.89 - 13) | <0.001 | 0.108 |

Values are no.(%) or median (IQR). ACEi = Angiotensin Converting Enzyme inhibitors; AF = Atrial Fibrillation; ARB = Angiotensin Receptor Blockers; ARNi = Angiotensin Receptor Neprilysin inhibitors; BMI = Body Mass Index; CCB = Calcium Channel Blockers; COPD = Chronic Obstructive Pulmonary Disease; CVA = Cerebro-Vascular Accident; DM = Diabetes Mellitus; eGFR = estimated Glomerular Filtration Rate; GLP-1 = Glucagon-Like Peptide-1; HFrEF = Heart Failure with reduced Ejection Fraction; HTN = Hypertension; IHD = Ischemic Heart Disease; IPTW = Inverse Probability Treatment Weighted; MRA = Mineralocorticoid Receptor Antagonist; SGLT2i = Sodium-Glucose coransporter-2 inhibitors; SMD = Standardized Mean Difference

Supplementary Table 2 – Echocardiographic parameters by significant TR:

|  | All (N=28,940) | No significant TR (N=24,897) | Significant TR (N=4,043) | p value |
| --- | --- | --- | --- | --- |
| Age, years | 75 (66 - 83) | 74 (65 - 82) | 80 (71 - 86) | <0.001 |
| **Female sex** | 12,420 (42.9%) | 10,103 (40.6%) | 2,317 (57.3%) | <0.001 |
| **BSA, m^2^** | 1.85 (1.7 - 2) | 1.86 (1.72 - 2.01) | 1.78 (1.63 - 1.93) | <0.001 |
| BMI, kg/m^2^ | 27.4 (24.3 - 31.3) | 27.6 (24.5 - 31.3) | 26.4 (23.4 - 30.4) | <0.001 |
| **Obesity (BMI ≥ 30 kg/m**^2^**)** | 8,448 (29.2%) | 7,487 (30.1%) | 961 (23.8%) | <0.001 |
| Heart rate, bpm | 75 (65 - 87) | 74 (65 - 86) | 78 (67 - 92) | <0.001 |
| SBP/DBP, mmHg | 130 (115 - 148) / 71 (63 - 80) | 131 (116 - 149) / 72 (63 – 80) | 125 (110 - 142) / 70 (61 – 80) | <0.001 |
| LVEDD, cm | 4.81 (4.34 - 5.40) | 4.84 (4.37 - 5.40) | 4.75 (4.24 - 5.32) | <0.001 |
| LVESD, cm | 3.2 (2.68 - 3.99) | 3.2 (2.69 - 3.96) | 3.2 (2.65 - 4.04) | 0.592 |
| LVEF, % | 55 (35 - 60) | 55 (35 - 60) | 55 (33 - 60) | <0.001 |
| Reduced LVEF (≤ 40%) | 10,316 (35.6%) | 8,835 (35.4%) | 1,481 (36.6%) | 0.163 |
| Septal thickness, cm | 1.11 (1 - 1.3) | 1.12 (1 - 1.3) | 1.1 (0.98 - 1.24) | <0.001 |
| LV mass index, g/m^2^ | 105 (85 - 127) | 105 (86 - 127) | 103 (84 - 125) | <0.001 |
| LA diameter, cm | 4.2 (3.78 - 4.66) | 4.1 (3.7 - 4.6) | 4.5 (4.1 - 5) | <0.001 |
| LA area, cm^2^ | 23.3 (19.7 - 28.0) | 23 (19 - 27) | 27 (23 - 32) | <0.001 |
| ≥ Moderate AS | 3,371 (11.6%) | 2,838 (11.4%) | 533 (13.2%) | <0.001 |
| ≥ Moderate MR | 4,276 (14.8%) | 2,781 (11.2%) | 1,495 (37.0%) | <0.001 |
| ≥ Moderate MS | 587 (2.0%) | 414 (1.7%) | 173 (4.3%) | <0.001 |
| Diastolic dysfunction ≥ grade 2 | 9,415 (32.5%) | 8,060 (32.4%) | 1,355 (33.5%) | 0.156 |
| sPAP, mmHg | 42 (33 - 52) | 40 (32 - 49) | 56 (48 - 66) | <0.001 |
| Elevated sPAP (sPAP ≥ 40 mmHg) | 13,774 (47.6%) | 10,097 (40.6%) | 3,677 (90.9%) | <0.001 |
| RV dysfunction | 4,379 (15.1%) | 2,749 (11.0%) | 1,630 (40.3%) | <0.001 |
| RA area, cm^2^ | 18 (14.7 - 23) | 17 (14 - 21) | 23 (19 - 28.2) | <0.001 |
| RA pressure, mmHg | 10 (5 - 10) | 10 (5 - 10) | 15 (10 - 20) | <0.001 |
| RV lead* | 2,435 (8.4%) | 1,801 (7.2%) | 634 (15.7%) | <0.001 |

Values are no.(%) or median (IQR). AS = Aortic Stenosis; BMI = Body Mass Index; BSA = Body Surface Area; DBP = Diastolic Blood Pressure; LA = Left Atrium; LVEDD = Left Ventricular End-Diastolic Diameter, LVEF = Left Ventricular Ejection Fraction; LVESD = Left Ventricular End-Systolic Diameter; MR = Mitral Regurgitation; MS = Mitral Stenosis; RA = Right Atrium; RV = Right Ventricle; SBP = Systolic Blood Pressure; sPAP = systolic Pulmonary Arterial Pressure; TR = Tricuspid Regurgitation

* RV lead from a pacemaker or implantable cardioverter-defibrillator insertion.

Supplementary Table 3 – Univariable and Multivariable Cox regression analyses for the primary outcome:

|  | Univariable | | | Multivariable | | |
| --- | --- | --- | --- | --- | --- | --- |
|  | HR | 95% CI | p Value | **HR** | 95% CI | p Value |
| Significant TR | 1.84 | 1.75 – 1.92 | <0.001 | 1.21 | 1.14 – 1.28 | <0.001 |
| Age | 1.06 | 1.06 – 1.06 | <0.001 | 1.05 | 1.05 – 1.05 | <0.001 |
| **Sex (Female)** | 1.25 | 1.15 – 1.30 | <0.001 | 0.95 | 0.91 – 0.99 | 0.025 |
| Obesity (BMI ≥ 30 kg/m^2^) | 0.87 | 0.83 – 0.90 | <0.001 | 0.96 | 0.91 – 1.0 | 0.073 |
| **HTN** | 1.51 | 1.46 – 1.56 | <0.001 | 1.03 | 0.98 – 1.08 | 0.2 |
| **DM** | 1.33 | 1.28 – 1.38 | <0.001 | 1.20 | 1.15 – 1.26 | <0.001 |
| **IHD** | 1.08 | 1.04 – 1.12 | <0.001 | 1.04 | 0.91 – 1.18 | 0.7 |
| **AF** | 1.49 | 1.43 – 1.55 | <0.001 | 0.96 | 0.92 - 1.01 | 0.1 |
| **COPD** | 1.82 | 1.72 – 1.93 | <0.001 | 1.61 | 1.51 – 1.72 | <0.001 |
| **CKD (eGFR < 45 mL/min/1.73m^2^)** | 2.15 | 2.07 – 2.24 | <0.001 | 1.43 | 1.37 – 1.50 | <0.001 |
| **Reduced LVEF (≤ 40%)** | 1.13 | 1.07 – 1.20 | <0.001 | 1.18 | 1.12 – 1.25 | <0.001 |
| **≥ moderate AS** | 1.21 | 1.15 – 1.26 | <0.001 | 1.05 | 0.99 – 1.11 | 0.11 |
| **≥ moderate MR** | 1.26 | 1.20 – 1.32 | <0.001 | 0.98 | 0.94 – 1.03 | 0.5 |
| **≥ grade 2 diastolic dysfunction** | 1.15 | 1.12 – 1.19 | <0.001 | 1.01 | 0.97 – 1.05 | 0.7 |
| Elevated sPAP (sPAP ≥ 40 mmHg) | 1.83 | 1.76 – 1.89 | <0.001 | 1.16 | 1.11 – 1.22 | <0.001 |
| RV dysfunction | 1.61 | 1.53 – 1.68 | <0.001 | 1.47 | 1.38 – 1.56 | <0.001 |
| SGLT2i | 0.54 | 0.50-0.59 | <0.001 | 0.62 | 0.56-0.68 | <0.001 |
| Loop diuretics | 1.89 | 1.82 – 1.96 | <0.001 | 1.23 | 1.18 – 1.28 | <0.001 |
| Thiazide diuretics | 1.23 | 1.17 – 1.30 | <0.001 | 1.04 | 0.98 – 1.10 | 0.2 |
| MRA | 1.24 | 1.19 – 1.29 | <0.001 | 1.08 | 1.03 – 1.14 | 0.003 |
| Beta blockers | 1.27 | 1.23 – 1.31 | <0.001 | 0.94 | 0.90 – 0.99 | 0.011 |
| ACEi/ARB | 1.32 | 1.28 – 1.36 | <0.001 | 0.95 | 0.91 – 0.98 | 0.006 |
| ARNi | 0.62 | 0.52 – 0.74 | <0.001 | 0.99 | 0.80 – 1.23 | >0.9 |
| GLP1-agonists | 0.79 | 0.69 – 0.91 | 0.001 | 0.89 | 0.76 – 1.04 | 0.15 |
| Serum Na | 1.0 | 0.99-1.0 | 0.064 | 0.99 | 0.99 - 1.0 | 0.032 |
| Serum K | 0.87 | 0.83 – 0.90 | <0.001 | 0.93 | 0.89 - 0.97 | <0.001 |
| Hemoglobin | 0.83 | 0.82 – 0.83 | <0.001 | 0.88 | 0.87 – 0.89 | <0.001 |

ACEi = Angiotensin Converting Enzyme inhibitors; AF = Atrial Fibrillation; ARB = Angiotensin Receptor Blockers; ARNi = Angiotensin Receptor Neprilysin inhibitors; AS = Aortic Stenosis; BMI = Body Mass Index; COPD = Chronic Obstructive Pulmonary Disease; DM = Diabetes Mellitus; eGFR = estimated Glomerular Filtration Rate; GLP-1 = Glucagon-Like Peptide-1; HTN = Hypertension; IHD = Ischemic Heart Disease; LVEF = Left Ventricular Ejection Fraction; MR = Mitral Regurgitation; MRA = Mineralocorticoid Receptor Antagonist; RV = Right Ventricle; SGLT2i = Sodium-Glucose coransporter-2 inhibitors; sPAP = systolic Pulmonary Arterial Pressure; TR = Tricuspid Regurgitation

Supplementary Table 4 – Baseline characteristics by SGLT2i use in the matched cohort:

|  | All (N=4,640) | No SGLT2i (N=2,320) | SGLT2i (N=2,320) | p value | SMD |
| --- | --- | --- | --- | --- | --- |
| Age, years | 71 (63 - 79) | 71 (62 - 79) | 72 (64 - 78) | 0.106 | 0.062 |
| **Female sex** | 1,394 (30%) | 690 (29.7%) | 704 (30.3%) | 0.677 | 0.013 |
| BMI, kg/m^2^ | 27.6 (25.2 - 31.8) | 27.7 (25.3 - 32.0) | 27.5 (25.1 - 31.6) | 0.207 | 0.027 |
| **Obesity (BMI ≥ 30 kg/m**^2^**)** | 1,599 (34.5%) | 811 (35%) | 788 (34%) | 0.497 | 0.021 |
| **HTN** | 2,792 (60.1%) | 1,395 (60.1%) | 1,397 (60.2%) | 0.976 | 0.002 |
| **DM** | 2,713 (58.5%) | 1,373 (59.2%) | 1,340 (57.8%) | 0.34 | 0.029 |
| **IHD** | 2,501 (53.9%) | 1,264 (54.5%) | 1,237 (53.3%) | 0.444 | 0.023 |
| **HFrEF** | 2,171 (46.8%) | 1,087 (46.9%) | 1,084 (46.7%) | 0.953 | 0.003 |
| **AF** | 1,144 (24.7%) | 548 (23.6%) | 596 (25.7%) | 0.109 | 0.048 |
| **COPD** | 363 (7.8%) | 182 (7.8%) | 181 (7.8%) | 1 | 0.002 |
| **CKD (eGFR < 45 mL/min/1.73m^2^)** | 748 (16.1%) | 359 (15.5%) | 389 (16.8%) | 0.247 | 0.035 |
| **CVA** | 725 (15.6%) | 354 (15.3%) | 371 (16%) | 0.518 | 0.020 |
| **Malignancy** | 396 (8.5%) | 181 (7.8%) | 215 (9.3%) | 0.083 | 0.052 |
| **Anti-coagulations** | 1,414 (30.5%) | 675 (29.1%) | 739 (31.9%) | 0.045 | 0.060 |
| **Insulin** | 1,216 (26.2%) | 608 (26.2%) | 608 (26.2%) | 1 | <0.001 |
| **Oral anti-diabetics** | 3,183 (68.6%) | 1,584 (68.3%) | 1,599 (68.9%) | 0.658 | 0.014 |
| **Beta blockers** | 3,601 (77.6%) | 1,813 (78.1%) | 1,788 (77.1%) | 0.398 | 0.026 |
| **Loop diuretics** | 3,417 (73.6%) | 1,732 (74.7%) | 1,685 (72.6%) | 0.125 | 0.046 |
| **Thiazide diuretics** | 437 (9.4%) | 219 (9.4%) | 218 (9.4%) | 1 | 0.001 |
| **MRA** | 1,543 (33.3%) | 780 (33.6%) | 763 (32.9%) | 0.618 | 0.016 |
| **ACEi/ARB** | 3,116 (67.2%) | 1,605 (69.2%) | 1,511 (65.1%) | 0.003 | 0.086 |
| **ARNi** | 309 (6.7%) | 117 (5.0%) | 192 (8.3%) | <0.001 | 0.130 |
| **GLP-1 agonists** | 419 (9.0%) | 205 (8.8%) | 214 (9.2%) | 0.682 | 0.014 |
| **CCB** | 1,454 (31.3%) | 733 (31.6%) | 721 (31.1%) | 0.782 | 0.011 |
| **Statins** | 3,381 (72.9%) | 1,674 (72.2%) | 1,707 (73.6%) | 0.291 | 0.032 |
| **Serum Na** | 139 (137 - 141) | 139 (137 - 141) | 139 (137 - 141) | 0.0962 | 0.043 |
| **Serum K** | 4.3 (4 - 4.6) | 4.3 (4 - 4.6) | 4.3 (4 - 4.6) | 0.396 | 0.020 |
| **Hemoglobin** | 12.3 (10.9 - 13.8) | 12.3 (10.9 - 13.7) | 12.3 (10.9 - 13.8) | 0.912 | 0.005 |

Values are no.(%) or median (IQR). ACEi = Angiotensin Converting Enzyme inhibitors; AF = Atrial Fibrillation; ARB = Angiotensin Receptor Blockers; ARNi = Angiotensin Receptor Neprilysin inhibitors; BMI = Body Mass Index; CCB = Calcium Channel Blockers; COPD = Chronic Obstructive Pulmonary Disease; CVA = Cerebro-Vascular Accident; DM = Diabetes Mellitus; eGFR = estimated Glomerular Filtration Rate; GLP-1 = Glucagon-Like Peptide-1; HFrEF = Heart Failure with reduced Ejection Fraction; HTN = Hypertension; IHD = Ischemic Heart Disease; IPTW = Inverse Probability Treatment Weighted; MRA = Mineralocorticoid Receptor Antagonist; SGLT2i = Sodium-Glucose coransporter-2 inhibitors; SMD = Standardized Mean Difference

Supplementary Figure 1 – Study flow chart:


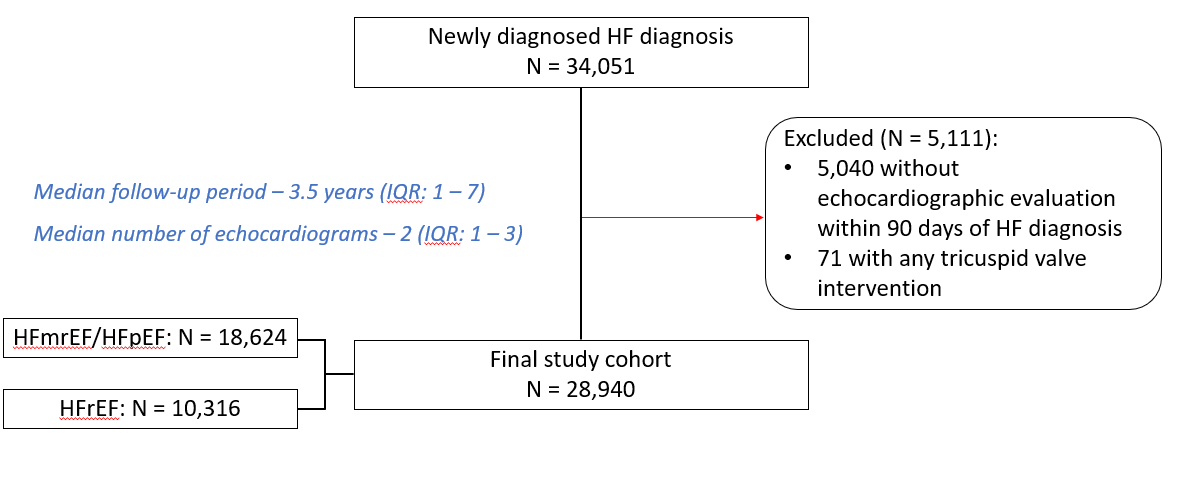


Supplementary Figure 1 legend:

The flow of participants showing the number of patients screened and excluded.

HF = Heart Failure; HFmrEF = HF with mildly-reduced Ejection Fraction; HFpEF = HF with preserved Ejection Fraction; HFrEF = HF with reduced Ejection Fraction; N = number of patients

Supplementary Figure 2 – Study flow chart:


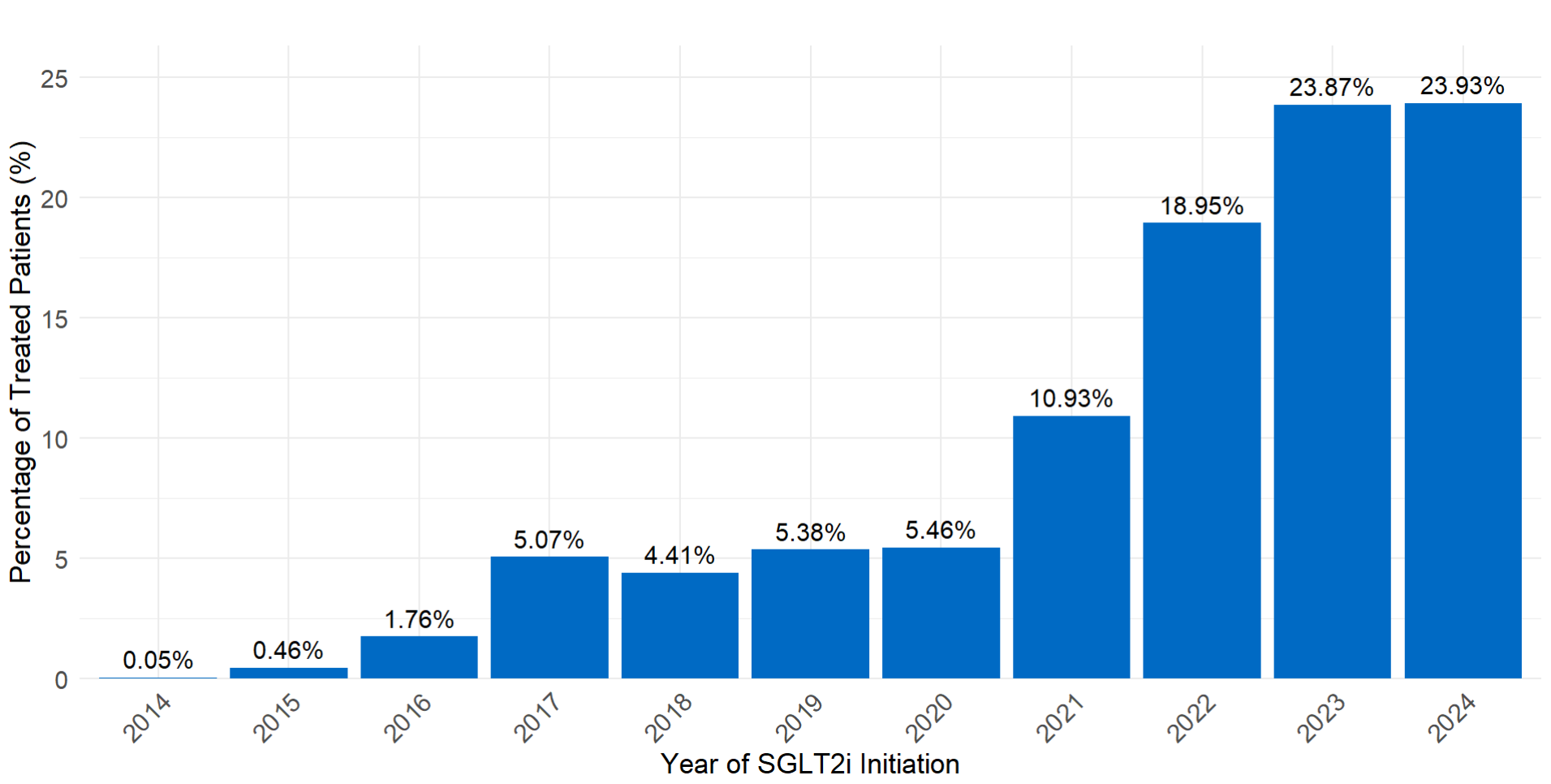


Supplementary Figure 2 legend:

Yearly uptake of SGLT2 inhibitors among the study population from 2014 to 2024. Bars represent the percentage of patients initiating SGLT2i in each calendar year. Percentages are displayed on top of each bar.

SGLT2i = Sodium-Glucose Cotransporter-2 inhibitors

Supplementary Figure 3 – Alluvial plot of the first and last TR severity grade stratified by SGLT2i use:


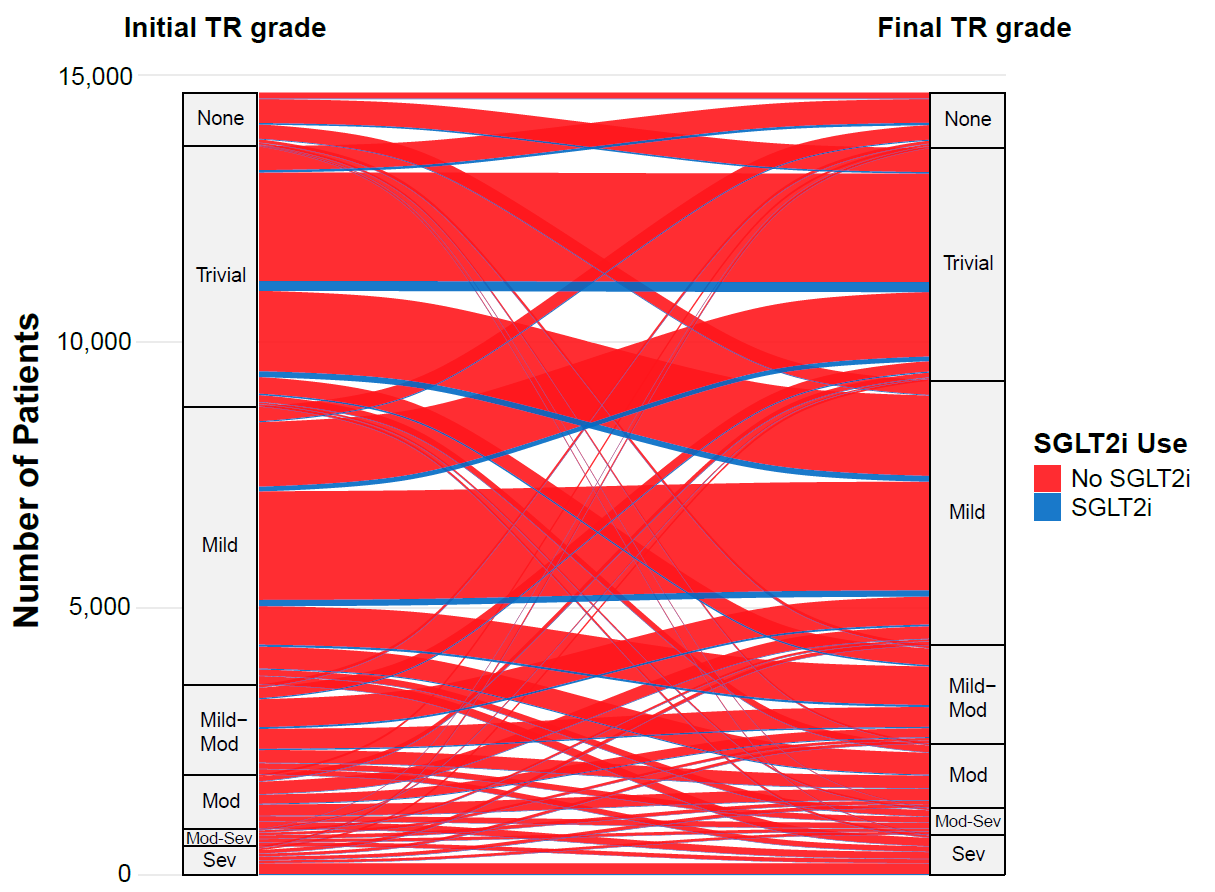


Supplementary Figure 3 legend:

This plot illustrates the changes in TR severity grade from the initial to the final evaluations, with colors indicating baseline SGLT2i use. Overall, TR progression of ≥2 grades occurred in 2,398 patients (16.3%), TR regression of ≥2 grades was observed in 461 patients (3.1%), and a change of ≤1 grade occurred in 11,820 patients (80.6%). Among patients using SGLT2i prior to the initial TR assessment (n = 899 [6.1%]), 10.1% experienced progression, 6.3% experienced regression, and 83.6% had no significant change. In patients who started SGLT2i during follow-up (n = 2,779 [18.9%]), 12.2% showed progression, 5.9% showed regression, and 81.9% had no significant change. Multivariable analysis treating SGLT2i use as a time-dependent variable demonstrated a 28% lower risk of TR progression by ≥2 severity grades (95% CI, 0.58–0.90; p < 0.001).

SGLT2i = Sodium-Glucose Cotransporter-2 inhibitors; TR = Tricuspid Regurgitation.

Supplementary Figure 4 – Box plot of the initial and final estimated sPAP stratified by SGLT2i use:


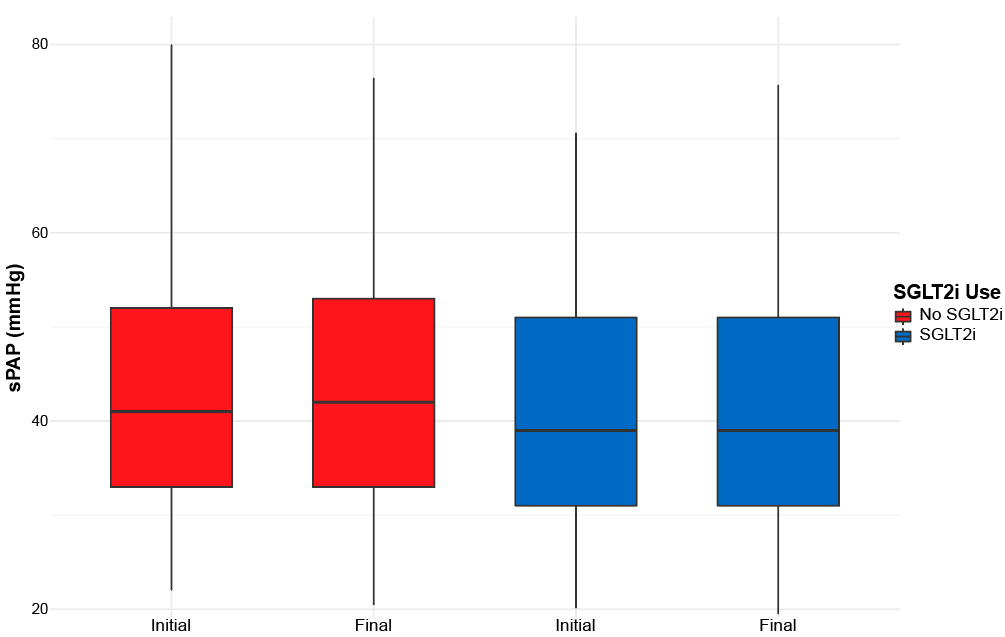


Supplementary Figure 4 legend:

This plot demonstrates the changes in estimated sPAP from the initial to the final evaluations, colored by SGLT2i use, demonstrating 36% lower risk of sPAP worsening in a multivariable model (95% CI 0.53–0.77; p<0.001).

SGLT2i = Sodium-Glucose Cotransporter-2 inhibitors; sPAP = systolic Pulmonary Arterial Pressure
